# A comparative analysis of pattern and attitude towards self-medication among pharmacy and non-pharmacy students in University of Ghana

**DOI:** 10.11604/pamj.2022.41.254.31013

**Published:** 2022-03-28

**Authors:** Seth Kwabena Amponsah, Gifty Odamtten, Ismaila Adams, Irene Akwo Kretchy

**Affiliations:** 1 Department of Medical Pharmacology, University of Ghana Medical School, Accra, Ghana,; 2 Department of Pharmacology and Toxicology, School of Pharmacy, University of Ghana, Accra, Ghana,; 3 Department of Pharmacy Practice and Clinical Pharmacy, School of Pharmacy, University of Ghana, Accra, Ghana

**Keywords:** Pharmacy, non-pharmacy, self-medication, college students, Ghana

## Abstract

**Introduction:**

self-medication involves the use of medicines without the input of health professionals. Available studies are not entirely conclusive on self-medication among health science versus non-health science university students. The current study therefore sought to investigate relevant aspects of self-medication among pharmacy and non-pharmacy students.

**Methods:**

this quantitative cross-sectional research was conducted among undergraduate pharmacy and non-pharmacy students of the University of Ghana from October 1^st^ 2019 to December 6^th^ 2019. Using a questionnaire, interviews were conducted to assess the pattern and attitude towards self-medication among respondents within the last 2 months.

**Results:**

a total of 337 (163 pharmacy and 174 non-pharmacy) students filled and completed questionnaires. The prevalence of self-medication was 55.2% for pharmacy and 51.1% for non-pharmacy students. Both pharmacy and non-pharmacy students were either accepting or ambivalent towards self-medication. Painkillers were the major class of medications that were self-medicated by both pharmacy (38.5%) and non-pharmacy students (30.7%). The most common reason for self-medication among pharmacy (62.2%) and non-pharmacy (56.2%) students was the need for rapid relief from an illness. Majority of the participants who were self-medicated (27.6% among non-pharmacy and 36.8% among pharmacy students) demonstrated ambivalent attitude towards self-medication. An increase in the study level reduced the likelihood of self-medication in both pharmacy and non-pharmacy students: OR=0.442, CI = 0.266-0.736 for pharmacy students and OR=0.671, CI = 0.456-0.987 for non-pharmacy students.

**Conclusion:**

self-medication is common students of the University of Ghana. Prevalence of self-medication was higher among pharmacy students than non-pharmacy students. This study provides data for targeted education and sensitisation of self-medication among university students.

## Introduction

Self-medication is the use of medicinal products without consultation of a medical practitioner. Self-medication is considered “responsible” when over-the-counter (OTC) medicines are administered at appropriate doses and at the specified duration [1]. Responsible self-medication is part of a self-care concept that empowers individuals to take responsibility for their health [2]. Nevertheless, self-medication may go beyond the use of OTCs, and may involve the use of prescription medications, herbal remedies, or traditional agents [3, 4]. The use of prescription-only medicines without the advice of a qualified health professional is referred to as irresponsible self-medication [5].

Despite the advantages, self-medication may lead to abuse of prescription-only medications, wrong self-diagnosis, and wrong drug dosing [6]. For example, in most resource-poor settings, antibiotics can be accessed without prescription [7]. While this behaviour indicates a lax in drug regulation [7], the practice of self-medication with antibiotics is a major contributor to antimicrobial resistance [8]. Reports suggest that self-medication may be high among young adults, which may include university students [2, 9]. The youth are particularly vulnerable to self-medication because of mass media exposure and advertisements of pharmaceuticals. Most youth or young adults patronize these pharmaceutical products without the advice of healthcare professionals. Some medications widely used by students for self-medication include analgesics, antimalarials, vitamins, antibiotics, anti-histamines, antacids, laxatives and anti-diarrheal agents [2, 6, 9]. Additionally, self-medication in this cohort of the population may be due to advice from friends, a feeling of having adequate knowledge of medicines (often via surfing the internet), or a feeling that their medical condition does not merit an appointment with a healthcare professional [9].

Previous studies have reported relatively high prevalence of self-medication among health science students in tertiary institutions. A study in South India reported self-medication in 78.6% of medical students [10]. Seam *et al*. [11], reported in their study that 88% of pharmacy students attested to practising self-medication. Other studies in Nigeria and United Arab Emirates among pharmacy students have indicated prevalence rates of 92.2% and 86%, respectively [12, 13]. The high incidence of self-medication among health science students may be due to information (efficacy and adverse effects) on drugs that is acquired as part of their study programs [11].

It is noteworthy, that studies available are not entirely conclusive on self-medication among health science versus non-health science students. A study conducted in Ghana by Donkor *et al*. [14], concluded that the prevalence of self-medication was lower in health science students in tertiary institutions compared to non-health science students. Conversely, a study conducted among students in Dubai concluded that the prevalence of self-medication was higher among health science students than non-health science students [15]. Previous studies mostly report the pattern of drugs used in self-medication; however, few have reported on attitude towards self-medication. The current study, therefore, sought to investigate pattern and attitude towards self-medication among pharmacy and non-pharmacy students. Findings from this study would provide data that would be used for targeted sensitisation on self-medication and its demerits among university students.

## Methods

**Study design and site:** this was an institution-based cross-sectional study conducted at the University of Ghana, Accra (Ghana), from October 1^st^ 2019 to December 6^th^ 2019. The University is the oldest and largest in Ghana, with a student population of about 37,940 as at the 2019/2020 academic year. University of Ghana is run on a collegiate system and comprises the following colleges: College of Basic and Applied Sciences, College of Education, College of Health Sciences and College of Humanities.

**Data source:** respondents of this study were pharmacy and non-pharmacy students of the University of Ghana. Non-pharmacy students were recruited from three randomly selected schools, namely, the University of Ghana Business School, School of Law and School of Agriculture. Pharmacy students were recruited from the School of Pharmacy.

**Eligibility criteria:** students in their first, second, third and fourth year undergraduate programs were included in the study. Students under the age of 18 were excluded because parental consent has to be sought prior to data collection.

**Sample size:** the minimum sample size was estimated based on the assumption of a 70% prevalence rate of self-medication [14]. Adopting a 95% confidence level, 5% margin of error, and a 10% non-responsive rate, the estimated sample size was 355. The sample size was split among pharmacy (n = 178) and non-pharmacy students (n = 177).

**Study tool:** a structured questionnaire adapted from previous studies was used [16, 17]. The questionnaire was tested for face validity by a panel of subject experts and modified in accordance with their recommendations to ensure comprehension by respondents. Internal consistency of the questionnaire was assessed using Cronbach's alpha and the scores ranged between 0.87-0.91. The questionnaire consisted of three sections; the first section required demographic characteristics of the respondents, the second part had close-ended questions on patterns of self-medication among respondents within the last 2 months and the third section measured attitudes towards medications (drugs) using the Beliefs about Medicines Questionnaire - specific option (BMQ-Specific). The BMQ-Specific comprises 10 items, with 5 items each for the Specific Necessity scale assessing the necessity of self-medications and Specific Concerns scale for assessing concerns about potential negative effects of self-medications [18].

**Data collection:** prior to data collection, the questionnaire was piloted among 20 students. The piloted study was conducted in the School of Education and Leadership which is in a different college (i.e. College of Education) from those where the study was conducted. As a result of this pilot study, some questions were modified to improve clarity. Sampling technique that was employed was purposive sampling. Students were approached at the end of their scheduled class to complete the questionnaire, which lasted approximately 20 minutes. Details of the study were shared with prospective respondents after written informed consent was obtained. Questionnaires were filled by each student under the guidance (or assistance) of the investigator.

**Ethics approval and consent to participate:** ethical approval for this research was obtained from the Institutional Review Board (IRB) of Noguchi Memorial Institute for Medical Research, University of Ghana (Approval number: 010/19-20). Informed consent was sought from all participants before administration of questionnaires. It was ensured that all information obtained from the questionnaire was kept confidential.

**Data analysis:** the data collected was cleaned, coded, stored, and analysed using Statistical Package for Social Sciences (version 20). Results were presented as percentages and in a tabular form. Using chi square, differences in variables (proportions) among pharmacy and non-pharmacy students were assessed. To analyse the effects of beliefs on self-medication, the sample was also divided into four groups according to the Necessity and Concerns scores (Sceptical, Indifferent, Ambivalent and Accepting). Group differences were assessed using an independent sample t test, chi-square or Fisher's exact test as appropriate. A stepwise backward logistic regression was used to predict whether a student would self-medicate or otherwise. Variables were selected for inclusion in the binary logistic regression modelling when significant. Odds ratios (ORs) with 95% confidence intervals (CIs) were used for observed associations. A p-value less than 0.05 was considered statistically significant.

## Results

**Demography:** out of the 355 participants eligible and approached for the study, 337 agreed to be part of the study, indicating a response rate of 95%. Of the 337 students (163 pharmacy and 174 non-pharmacy students) who completed the questionnaire, 52.2 % of them were female. A majority of the respondents were between 18 - 20 years. A summary of demographic characteristics of respondents is shown in Table 1.

**Table 1:** demographic data of respondents (n = 337)

Demographic information	Frequency	Percentages
**Age**		
18-20	244	72.4
21-23	86	25.5
24-41	7	2.1
**Sex**		
Male	161	47.8
Female	176	52.2
**Programme of study**		
Non-pharmacy	174	51.6
Pharmacy	163	48.4
**Year of study**		
Year 1	84	24.9
Year 2	86	25.5
Year 3	91	27.0
Year 4	76	22.6

**Prevalence of self-medication among students:** from data collected, 179 out of 337 (53.1%) of the respondents (pharmacy and non-pharmacy students) practised self-medication. Prevalence of self-medication was higher among pharmacy than non-pharmacy students. However, the difference was not statistically significant (p = 0.455); as shown in Table 2. Data also showed that 39 (48.8%) male and 50 (52.6%) female non-pharmacy students self-medicated. Among pharmacy students, 40 (49.4%) males and 50 (61.7%) females engaged in self-medication.

**Table 2:** prevalence of self-medication among pharmacy and non-pharmacy students, medicines commonly used, reasons and sources of medication used, actions taken in response to treatment failure and side effects observed by students

	Non-Pharmacy		Pharmacy		Total	p value
	(n)	(%)	(n)	(%)		
Students who self-medicated	89	51.1	90	55.2	179	0.455
Students who did not self-medicate	85	48.9	73	44.8	158	
**Class of drugs**						
Antibiotics	19	10.6	11	6.1	30	0.124
Sedatives	7	3.9	5	2.8	12	0.567
Vitamins	15	8.4	19	10.6	34	0.478
Herbal products	7	3.9	5	2.8	12	0.567
Anti-allergy medication	8	4.5	8	4.5	16	1.000
Antacids	9	5.0	14	7.8	23	0.280
Cough syrup	8	4.5	10	5.6	18	0.635
Anti-diarrheal medication	6	3.4	7	3.9	13	0.801
Painkillers	55	30.7	69	38.5	124	0.121
Cannot remember	4	2.2	3	1.7	7	0.733
Others	4	2.2	6	3.4	10	0.492
**Reasons**						
Presence of long hospital queues	20	22.5	18	20.0	38	0.684
For fast relief of illness	50	56.2	56	62.2	106	0.415
Illness was not severe	37	41.6	40	44.4	77	0.706
Hospital fees are expensive	3	3.4	1	1.1	4	0.300
Lack of trust in health practitioners	0	0.0	2	2.2	2	0.161
For emergency purposes	17	19.1	20	22.2	37	0.610
Previous medicine use	32	36.0	36	40.0	68	0.583
Others	0	0.0	1	1.1	1	0.323
**Sources of drugs**						
Community Pharmacy	56	62.9	71	78.9	127	0.019*
Licensed chemical sellers	36	40.4	18	20.0	54	0.003*
Leftover medicines from previous use	12	13.5	17	18.9	29	0.328
Supermarkets	2	2.2	2	2.2	4	1.000
Others	3	3.4	3	3.3	6	0.971
**Actions taken in treatment failure**						
Treatment was suspended	25	28.1	30	33.3	52	0.452
Treatment was repeated	17	19.1	10	11.1	27	0.219
Help was sought from a health professional	55	61.8	66	73.3	121	0.101
Others	2	2.2	1	1.1	3	0.564
**Side effects**						
Stomach pains	3	3.4	4	4.4	7	0.565
Vomiting	1	1.1	2	2.2	3	0.524
Fever	3	3.4	1	1.1	4	0.300
Diarrhea	0	0.0	5	5.6	5	0.024*
Headache	3	3.4	4	4.4	7	0.731
Dizziness	5	5.6	7	7.8	12	0.557
Others	1	1.1	3	3.3	3	0.318

* indicates p < 0.05

**Patterns of medicines used and self-medication behaviour:** the most self-medicated class of drugs among non-pharmacy students was painkillers (30.7%). Painkillers were also the most self-medicated drugs among pharmacy students (38.4%). A comparison of students using painkillers: pharmacy versus non-pharmacy students showed that the difference did not attain statistical significance (p = 0.121). Antibiotics were also found to have been used by students; 10.6% non-pharmacy and 6.1% pharmacy, respectively. A summary of medicines self-medicated by students is shown in Table 2. Most (62.2%) pharmacy students who self-medicated mentioned fast relief of illness as the reason for self-medication. Also, most (56.2%) of the non-pharmacy students mentioned fast relief of illness as reason for self-medication. Other reasons for self-medication among the students are shown in Table 2.

Most of the respondents stated that they had obtained their medicines from community pharmacies; 78.9% of pharmacy students and 62.9% of non-pharmacy students, respectively. More non-pharmacy students (40.4%) derived their medicines from licensed chemical sellers as compared to pharmacy students (20.0%). Other sources of drugs used in self-medication are summarized in Table 2. Many of the respondents indicated that they sought help from health professionals when self-medication failed. The aforementioned action was reported in 61.8% of non-pharmacy and 73.3% of pharmacy students, respectively. Other actions taken by students who self-medicated and treatment failed are shown in Table 2. Some of the students who self-medicated reported side effects. A list of some side effects reported by respondents is shown in Table 2.

**Attitude towards self-medication using BMQ Specific version:** categorization of students into four attitudinal groups found that for both pharmacy and non-pharmacy students, most of the students were in the Accepting or Ambivalent groups. However, self-medication among the students did not vary significantly between the four groups (Fisher's exact test, p = 1). Table 3 summarizes the various groups and their respective percentages.

**Table 3:** effects of attitude on self-medication among pharmacy and non-pharmacy students

Attitude	Non-Pharmacy		Pharmacy		Total	p value
	(n)	(%)	(n)	(%)		
**Self-medicated in the past two months**						
Skeptical	10	5.7	5	3.1	15	0.584
Indifferent	7	4.0	9	5.5	16	0.237
Ambivalent	48	27.6	60	36.8	108	0.480
Accepting	24	13.8	16	9.8	40	0.584
**Did not self-medicate in past two months**						
Skeptical	6	3.4	4	2.5	10	0.584
Indifferent	14	8.0	7	4.3	21	0.237
Ambivalent	40	23.0	43	26.4	83	0.480
Accepting	25	14.4	19	11.7	44	0.584

**Predicting the likelihood of self-medication among students using logistic regression models:** the models correctly predicted 86% of cases for pharmacy students and 85% of cases for non-pharmacy students. The models showed inverse associations between the predictive variables and self-medication. An increase in the study level reduced the likelihood of self-medication in both pharmacy and non-pharmacy students: OR=0.442, CI = 0.266-0.736 for pharmacy students and OR=0.671, CI = 0.456-0.987 for non-pharmacy students. Study level, and students who self-medicated with painkillers were common to both groups. A summary of the variables used in the models are shown in Table 4.

**Table 4:** logistic regression model predicting self-medication among students

	Wald	p-value	OR	95% C.I. for OR
**Pharmacy students**				
Study Level	9.841	0.002	0.442	0.266-0.736
Self-medicated with Pain killers	29.907	0.000	0.002	0.000-0.021
**Non-Pharmacy students**				
Study Level	4.101	0.043	0.671	0.456-0.987
Self-medicated with Pain killers	35.691	0.000	0.021	0.006-0.075
Self-medicated with Antibiotics	10.277	0.001	0.031	0.004-0.259
Self-medicated with Anti-allergy drugs	4.354	0.037	0.088	0.009-0.863

OR: Odds ratio, CI: Confidence interval

## Discussion

With contrasting studies on self-medication among health science and non-health science students, the current study sought to assess self-medication among pharmacy and non-pharmacy students in the University of Ghana. Prevalence of self-medication among the entire population (pharmacy and non-pharmacy students) was found to be 53.1%. This means nearly half of the students attested to self-medicating within the last 2 months. However, a number of studies have reported higher prevalence of self-medication; 62.9% among students in Egypt [9], and 96.8% in a Jordanian University [19]. The differences in prevalence in the aforementioned studies and current study may be due to varying demography of respondents, socio-economic variables and study methods [20].

The current study showed that pharmacy students (55.2%) self-medicated more than non-pharmacy students (51.1%), although the difference was not statistically significant. There are a number of studies that have demonstrated that self-medication is primarily practised by health science students [16, 21]. Self-medication being greater among pharmacy students than non-pharmacy students in the current study is similar to a study in Egypt, which found self-medication to be higher among medical students [22]. It may be possible that pharmacy students´ knowledge derived from their program of study influenced their self-medication practices. A study conducted among medical students in India suggested that medical students had a strong predisposition to self-medication because they had ample knowledge in pharmacology [10].

In previous studies, medications widely used by students for self-medication included analgesics, antimalarials, vitamins, antibiotics, anti-histamines, nasal decongestants, antacids, laxatives, anti-diarrheal and anti-emetics [13, 23]. In this study, painkillers were the most used class of drugs for self-medication by both pharmacy (38.5%) and non-pharmacy (30.7%) students. Other studies conducted among students have also found painkillers as commonly used self-medication agents [20, 24]. Most illnesses or disorders usually present with pain [19], as such pain-relieving agents are often used. Students in this study also reported using antibiotics. Irrational use of antibiotics without appropriate diagnosis contributes to increase health-care cost, high morbidity, and the emergence of resistant strains of infectious microorganisms [8].

The logistic regression model sought to explain the possible predictors of self-medication among the students. For both pharmacy and non-pharmacy students, an increase in study level showed a decrease in the likelihood to self-medicate. This finding was contrary to a similar study in Nigeria [25], and could be because, with every increase in study level, students became more knowledgeable on the rational use of drugs. This decline was more pronounced in pharmacy students compared with non-pharmacy students. For non-pharmacy students, other predictors of the likelihood to self-medicate were students who self-medicated with antibiotics and anti-allergy drugs. This finding could suggest that students who self-medicated with pain relievers, anti-allergy drugs and antibiotics were more likely to self-medicate again in the next 2 months.

Data from the present study showed that the main reason for self-medication among pharmacy students (62.2%), as well as non-pharmacy students (56.2%) was rapid relief of illness. The other reason for self-medication among these students was that they perceived the illness as not being severe. There is the likelihood that students may self-medicate in order to avoid missing out on lectures and other academic activities; as seen among female students who use painkillers during periods of dysmenorrhea. Our findings corroborate studies conducted by Kumar *et al*. [10], and Gelayee [20], who also found that students self-medicated because they felt that the disease was too trivial to warrant a hospital visit.

Data from this study showed that community pharmacies were the main sources of drugs for self-medication by the students. Non-pharmacy students (40.4%) were also found to patronize OTCMS. A few students from both groups also obtained drugs from supermarkets. In Ghana, medicines are rarely sold in supermarkets, and this may have accounted for the low level of patronage in this report. It is noteworthy, however, that there are a number of community pharmacies on the University of Ghana campus, making that a convenient source of drugs for students.

A number of students who self-medicated in the current study reported some side effects, such as diarrhoea and dizziness. For rational self-medication, an individual must have adequate information on possible side effects [23]. Individuals may be prone to risks associated with self-medication, especially if they do not have adequate knowledge in identifying symptoms of a disease and/or choose the right drug (and dose) for a disease condition [26]. Additionally, the majority of the participants who self-medicated (27.6% among non-pharmacy and 36.8% among pharmacy students) demonstrated ambivalent attitudes towards self-medication. Knowledge has an intrinsic relationship with attitudes, which precedes intention. It is, thus, not surprising that respondents had ambivalent attitudes towards self-medication. The current study had some limitations. Recall bias could have affected some responses from participants. In addition, cause and effect relationship between variables was not established.

## Conclusion

From the current study, prevalence of self-medication was found to be 53.1% among all students interviewed. The prevalence of self-medication was higher among pharmacy students than non-pharmacy students. Painkillers were found to be the most self-medicated class of drugs in this study. The majority of the participants who self-medicated (27.6% among non-pharmacy and 36.8% among pharmacy students) demonstrated ambivalent attitudes towards self-medication. An increase in the study level reduced the likelihood of self-medication among respondents. While this study provides data for targeted education and sensitisation of self-medication among university students, health professionals have to also improve awareness of students about use of prescription-only drugs such as antibiotics and its health consequences. Regulatory and healthcare authorities have a major role to play in restricting availability of especially prescription-only drugs to the populace.

### What is known about this topic

- Self-medication may be high among young adults, which may include university students;
- There is contrasting data on the prevalence of self-medication among health science and non-health science students in the university.

### What this study adds

- Majority of the participants who self-medicated (non-pharmacy and pharmacy students) demonstrated ambivalent attitudes towards self-medication;
- An increase in the study level reduced the likelihood of self-medication among non-pharmacy and pharmacy students.
